# Complementary and Alternative Medicine: A Survey of Its Use in Pediatric Oncology

**DOI:** 10.1155/2013/527163

**Published:** 2013-11-06

**Authors:** Rafiaa Valji, Denise Adams, Simon Dagenais, Tammy Clifford, Lola Baydala, W. James King, Sunita Vohra

**Affiliations:** ^1^Department of Pediatrics, University of Calgary, Calgary, AB, Canada T2N 4N1; ^2^CARE Program, Department of Pediatrics, University of Alberta, Edmonton, AB, Canada T5K 0L4; ^3^Palladian Health, West Seneca, NY 14224, USA; ^4^Departments of Pediatrics, and of Epidemiology & Community Medicine, University of Ottawa, Ottawa, ON, Canada K1N 6N5; ^5^Canadian Agency for Drugs and Technologies in Health, Ottawa, ON, Canada K1S 5S8; ^6^Department of Pediatrics, Faculty of Medicine and Dentistry, University of Alberta, Edmonton, AB, Canada T6G 2R3; ^7^Division of Pediatric Medicine, Department of Pediatrics, University of Ottawa, Ottawa, ON, Canada K1N 6N5; ^8^Children's Hospital of Eastern Ontario, Ottawa, ON, Canada K1H 8L1; ^9^Department of Pediatrics, Faculty of Medicine & Dentistry and School of Public Health, University of Alberta, 8B19-11111 Jasper Ave, Edmonton General Hospital, Edmonton, AB, Canada T5K 0L4

## Abstract

*Background.* The use of complementary and alternative medicine (CAM) is high among children and youths with chronic illnesses, including cancer. The objective of this study was to assess prevalence and patterns of CAM use among pediatric oncology outpatients in two academic clinics in Canada. *Procedure.* A survey was developed to ask patients (or their parents/guardians) presenting to oncology clinics at the Stollery Children's Hospital in Edmonton and the Children's Hospital of Eastern Ontario (CHEO) in Ottawa about current or previous use of CAM products and practices. *Results.* Of the 137 families approached, 129 completed the survey. Overall CAM use was 60.5% and was not significantly different between the two hospitals. The most commonly reported reason for not using CAM was lack of knowledge about it. The most common CAM products ever used were multivitamins (86.5%), vitamin C (43.2%), cold remedies (28.4%), teething remedies (27.5%), and calcium (23.0%). The most common CAM practices ever used were faith healing (51.0%), massage (46.8%), chiropractic (27.7%), and relaxation (25.5%). Many patients (40.8%) used CAM products at the same time as prescription drugs. *Conclusion.* CAM use was high among patients at two academic pediatric oncology clinics. Although most respondents felt that their CAM use was helpful, many were not discussing it with their physicians.

## 1. Introduction 

Complementary and alternative medicine (CAM) consists of a diverse group of medical and healthcare systems, products, and practices that are considered to be outside of conventional medicine, including nutritional supplements, vitamins, herbal remedies, diet changes, spiritual therapy, chiropractic, osteopathy, yoga, homeopathy, massage, acupuncture, and aromatherapy [1]. In children, CAM has been used for a variety of chronic illnesses including asthma [2–4], arthritis [5], gastrointestinal diseases [6–10], and neurological or developmental disorders [11–14]. Within the pediatric oncology population, CAM use has been reported as high as 84% [15–17], most commonly in a complementary fashion (i.e., alongside conventional care) [18]. 

 Pediatric CAM use has been associated with parental CAM use, poor prognosis of the child, and increased parental age or education [15]. Reasons reported for CAM use are varied, including a desire to explore all possible treatment options, enhance the efficacy or minimize side effects of conventional therapy, boost immunity, cure the cancer or slow its progression, and increase feelings of control over the child's treatment [18–20]. Many patients describe CAM as being helpful, and few report adverse effects [18–20]. Despite the popularity of CAM, only half of parents disclose their child's CAM use to their physicians [16]. Less than half of pediatric oncologists inquire about CAM use, most often due to lack of time and knowledge or discomfort due to concern over harmful side effects [21]. 

The purpose of this study was to examine issues around CAM use by pediatric oncology outpatients in two clinics in Canada. Issues explored expanded on the limited information available from reports in other pediatric oncology populations (including 2 in other areas of Canada and 1 in the US) such as rates and reasons for CAM use, reporting of use to healthcare practitioners, and safety of use, as well as novel areas including reasons for nonuse of CAM, sources of CAM information and trust in these sources, and concurrent use of CAM and conventional care, including specific drug/CAM combinations. 

## 2. Methods 

This paper is part of a larger study that was carried out at the Stollery Children's Hospital in Edmonton, Alberta, Canada, and the Children's Hospital of Eastern Ontario (CHEO) in Ottawa, Ontario, Canada. Full methods are available in Adams et al. 2013 [22]. Five pediatric subspecialty outpatient clinics were chosen for the larger study (cardiology, gastrointestinal, neurology, oncology, and respiratory). Patients in these subspecialties were surveyed at each of the two locations. These subspecialties were chosen because they see many patients with chronic conditions who may use CAM practices and/or products. The survey contained 19 questions that examined patient and family demographics (such as age, gender, family income, parent education, time since diagnosis, etc.), general health (ranked from poor to excellent), use of specific CAM products and therapies, reasons for use or nonuse, concurrent use with conventional medicine, disclosure about use, satisfaction with care and adverse effects, or CAM. Children and/or their families were eligible to participate in this study if they had not previously filled out a survey, were under 18 years of age, and could read French or English. Parents/caregivers completed the surveys for younger children and in conjunction with older children as appropriate.

 Data was entered into a database (SPSS 11). Descriptive statistics were tabulated as percentages for categorical variables and means (standard deviation) or medians (IQR) for continuous scaled variables and numbers. Participant demographics, general health and use of specific CAM products and practices, satisfaction with care, and beliefs about CAM were compared by centre (Stollery vs. CHEO) using chi-square tests, Wilcoxon tests, and independent *t*-tests as appropriate. 

The use of CAM was compared between the two sites and modelled by univariate and multivariate logistic regression. Differences in sample sizes between the sites were taken into account. Predictor variables included child's gender, age, ethnicity, health status, and time since diagnosis as well as family's use of CAM, family's CAM insurance, parent's education and income, and discussion of CAM with conventional medical practitioner. Regression diagnostics such as *c*-statistics, *r* square, and Hosmer and Lemeshow lack of fit statistics were carried out. Measures for influential observations and detecting outliers were likewise considered. 

## 3. Results

Of 137 families approached, 112 from the Stollery in Edmonton and 25 from CHEO in Ottawa, we obtained a sample size of 129 pediatric oncology patients (*n* = 107 from Edmonton and *n* = 22 from Ottawa). Four of the families approached refused to participate, and, for another 4, the patients were 18 years or older.

### 3.1. Population Characteristics

The pediatric oncology population sampled consisted of 55% males with a mean age of 9 years. The majority of patients reported their ancestry as Caucasian (50.0%) or Canadian/French Canadian (33.1%), with others self-described as First Nations/Inuit/Metis (14.5%), South Asian (8.1%), East Asian (1.6%), Middle Eastern/Arabic (2.4%) and Latin American or Mexican (0.8%). The self-reported health status of most patients ranged from good to excellent (85.1%) rather than fair or poor (14.9%). Most users (61.2%) had received their diagnosis more than 12 months earlier. Reported CAM use in Edmonton was not significantly different than that in Ottawa (62.6% vs. 50.0%, resp.; *P* > 0.05). Within each site, CAM use was not significantly associated with the child's age, gender, ancestry, reported health status, time of diagnosis, or insurance coverage (Table 1).

The caregiver population had a mean age of 38.5 years and consisted mostly of primary caregivers (94.5%), most commonly mothers (73.6%), who believed themselves to be knowledgeable about their child's use of CAM. The health status of most caregivers ranged from good to excellent (98.5%). A significantly greater proportion of Ottawa caregivers (45.0%) had completed secondary school as their highest level of education than Edmonton caregivers (22.1%, *P* = 0.03). The caregiver populations did not differ significantly in terms of annual household income, with the majority (77.8%) of families reporting a household income of over $40,000 annually. Approximately two thirds of caregivers reported that they had ever used CAM for themselves (Table 1).

Caregiver CAM use was associated with 8.7 greater likelihood (*P* < 0.001) of child CAM use in Edmonton and 17.5 times in Ottawa (*P* = 0.02). Within both populations, parental education and income were not associated with child CAM use. 

### 3.2. Products/Practices

The most common CAM products ever used or currently used were multivitamins (86.5%, 71.7%) and vitamin C (43.2%, 32.6%), respectively, while the most common CAM practices used were faith healing (e.g., praying for one's own health or having others pray for one's health [23] (51.1%, 50.0%) and massage (46.8%, 38.9%), respectively. Patients who used only multivitamins/minerals accounted for 13% of respondents. The majority of products and practices were found to be helpful by the majority of users (Table 2). 

Common reasons for children or caregivers not using CAM, respectively, included lack of knowledge about CAM (49.0%, 50.0%), not thinking it was necessary (25.5%, 28.9%), and, for children with cancer, concern about side effects from combining CAM with conventional treatments (27.5%, 5.3%). 

### 3.3. Safety Issues

In general, half of users (50.8%) reported using any type of CAM concurrently with conventional medicine, while 15.9% reported using CAM before trying conventional medicine; 40.8% reported using CAM with prescription drugs. The most common combinations were vitamins/minerals taken in conjunction with immune suppressants or antibiotics (Table 3). 

 A total of seven adverse effects were reported and of these, four were rated as mild by respondents. One moderate adverse effect was reported for each of multivitamins, cold remedy, and acupuncture; no severe effects were reported. No further adverse effect data were provided. 

### 3.4. Information Sources

Sources commonly used for CAM information by pediatric oncology patients/caregivers included families (66.7%), CAM providers (37.7%), health food stores (37.7%), the pediatric oncology clinic (33.3%), pharmacies (33.3%), books/magazines (30.4%), other healthcare providers (26.1%), and the Internet (26.1%). 

 The most trusted sources, as scored on a 10-point Likert scale where 1 indicated no trust and 10 indicated complete trust, included the pediatric oncology clinic (8.7), CAM providers (8.7), pharmacies (7.9), family (7.9), other healthcare providers (7.6), and health food stores (7.4). The Internet (5.7) and television (5.3) were trusted the least. Pharmacies were significantly more trusted by CHEO than Stollery patients (*P* = 0.03). 

The majority (76.7%) of those who used CAM concurrently with prescription drugs reported consulting their physician about this use, while 57.1% consulted a pharmacist. The majority of caregivers either “agreed” or “strongly agreed” that they felt comfortable discussing CAM in the pediatric oncology clinic (70.2%), and approximately half (49.6%) would have liked more information about CAM from the clinic. They also reported they would be more likely to use CAM products (53.7%) and practices (55.4%) if they were available in the clinic. 

## 4. Discussion

Our work confirms that CAM use by pediatric oncology patients is highly prevalent (15–20) and is perceived by patients to be beneficial (24, 25) and harmless (19). Additionally, we present novel information about reasons for nonuse of CAM, sources of CAM information, and trust in these sources, as well as concurrent use of CAM and conventional care, including specific drug/CAM combinations. 

An alarmingly high proportion of patients, anywhere from 41% [23] to 92% [24], do not discuss CAM use with their physicians. Patients fail to initiate CAM discussions because they perceive them as irrelevant and are afraid of physician discouragement or due to prior lack of physician inquiry [25]. Optimistically, the majority of our population stated they were comfortable discussing CAM in the clinic and high proportion reported consulting their doctor about concurrent use of CAM with prescription drugs; however, a gap remains between use and disclosure, and we recommend that health care providers routinely inquire about CAM use as part of their history taking. Our data confirm use can change over time, and as it is a dynamic phenomenon, reinquiry is recommended at each health visit. 

The most popular therapies continue to be CAM products, also known as natural health products (NHPs), especially multivitamins [26, 27]. As others have reported, the most popular CAM therapy sought by pediatric oncology patients was faith healing [28, 29]. Patients who do not use CAM feel they do not know enough about it, including potential side effects from combining CAM with conventional treatments. Potential interaction between CAM and conventional therapies is a valid concern, as illustrated by the ongoing debates about the role of NHPs with antioxidant or immune modulator properties [30–45]. 

Simultaneous use of multiple complementary therapies [20] with conventional treatments [18, 46] continues to be prevalent and can lead to clinically relevant drug interactions. These interactions are especially concerning given the narrow therapeutic range of most anticancer drugs [47] and the increased susceptibility of the seriously ill to adverse drug effects [48]. NHP-drug and NHP-NHP interactions can be pharmacokinetic or pharmacodynamic, often involving changes in expression or function of the hepatic, extra-hepatic, and potentially tumour-expressed CYP 450 enzymes, which metabolize the majority of anticancer drugs and ATP efflux pumps such as P-glycoprotein, which are involved in drug transport [47, 49–51]. Multivitamins, the most common CAM product used concurrently with prescription medications in our study, have been shown to inhibit CYP 450 metabolism in vitro; this may explain their frequent implication in interactions with other natural health products and conventional treatments [52]. Immune modulators, such as Echinacea and ginseng, were also commonly used by patients in our study. Given that cancer itself and conventional treatments are immunosuppressive, it is understandable that patients use herbal supplements to help fight infection and the cancer, similar to conventional use of colony stimulating factors [42].

Of greatest potential concern is the lack of discussion/disclosure about CAM use by patients. Conventional health care providers may not ask because of discomfort, lack of familiarity, or education about CAM during their training (21). Given our findings that patients/families have a high degree of trust in their oncology clinics and prefer them as a source of reliable information about CAM, there is a need for change. Patient-centred care demands that pediatric oncology clinics acknowledge this gap and address it by creating opportunities for families to ask questions about popular CAM therapies. This would be preferable to families having to search for information on the internet or at health food stores, since the quality of this information is suspected, including potential financial conflicts of interests [53, 54]. While CAM providers are expert in their discipline, few have specific pediatric training or knowledge of the specialized health needs of oncology patients [55]. It may therefore be difficult for them to anticipate or respond to pediatric oncology patient needs without greater communication with the oncology team that cares for the patient. 

The majority of respondents wanted to discuss CAM with their pediatric oncology clinic and were more likely to use CAM products and practices if they were available in clinic. In response to this kind of patient demand, integrative oncology is a new and growing field. Pediatric integrative oncology centers are starting to emerge and bring to light new and important issues that must be considered when offering CAM therapies including legal, ethical, and clinical issues [54, 56].

Our study is limited by dependence on recall of past events by a proxy (parent/caregiver) response; parents, however, are often asked to comment on various aspects of their child's health, as occurs at annual medical checkups. In addition, recent evidence suggests that recall of regularly consumed natural health products, as measured by a single questionnaire, is comparable to more detailed methods (i.e., diary) [57]. Because CAM use varies between ethnic groups [58–62] and our survey was only administered in English or French, our findings may be limited in their generalizability. An additional limitation is the inclusion of only two clinics. By sampling in two geographically and socioeconomically distinct regions, we were able to add to existing knowledge about CAM use in pediatric oncology centers. Given the very high rates of use described in this population, further sampling is warranted. 

Future research directions include sampling in other regions, including translation to facilitate participation by other ethnic groups. Particular emphasis should be paid to “best cases,” that is, pediatric oncology patients who experience better-than-expected outcomes in association with their CAM use [63], as well as those who suffer serious adverse events [64]; both offer valuable lessons from which the field can learn and grow. Investigation of NHP-drug interactions in oncology patients is important to pursue, so that future counselling can help guide patients on which NHP-drug combinations may be safe and which should be avoided. Finally, CAM use in survivors of childhood cancer is another intriguing area that is worthy of further consideration. 

## 5. Conclusions

CAM use within the pediatric oncology population continues to be highly prevalent and is associated with caregiver use, necessitating routine inquiry by physicians. By far, the most commonly reported source of CAM information is the family. To ensure that patients use evidence-based information of the highest quality in their decision making so as to maximize efficacy and ensure safety, pediatric oncology clinics and healthcare providers need to play a greater role. The concurrent use of CAM with conventional treatment by about half of pediatric oncology patients requires that their physicians actively prevent, monitor, and report adverse effects. Pediatric oncology has made tremendous strides through the systematic capture of data to drive the next generation of treatment options; there is an urgent need to include patient CAM use in this data registry, to identify which therapies are helpful and which are harmful, and to communicate these finding to patients and their families. These findings may be beneficial to existing CAM programs in pediatric oncology as well as those considering new programs.

## Figures and Tables

**Table 1:** Demographic information.

	*n*	Edmonton	*n*	Ottawa	Total
*Patient Information *					
Child/youth age mean (SD)	107	8.9 (4.4)	22	9.0 (4.8)	8.9 (4.5)
Gender	107		22		
Female *N* (%)		45 (42.1)		13 (59.1)	58 (45.0)
Time since diagnosis	107		22		
0–3 mo.		15 (14.0)		5 (22.7)	20 (15.5)
3–6 mo.		11 (10.3)		1 (4.5)	12 (9.3)
6–12 mo.		15 (14.0)		3 (13.6)	18 (14.0)
>12 mo.		66 (61.7)		13 (59.1)	79 (61.2)
If child/youth has ever used CAM	107		22		
Yes		67 (62.6)		11 (50.0)	78 (60.5)
*Parent/Caregiver Information *					
Age mean (SD)	104	38.5 (8.7)	22	38.5 (7.2)	38.5 (8.4)
Gender	107		22		
Female *N* (%)		81 (75.7)		17 (77.3)	98 (76.0)
Highest completed level of education	104		20		
No formal education		0		0	0
Primary school only		3 (2.9)		1 (5.0)	4 (3.2)
Secondary school*		23 (22.1)		9 (45.0)	32 (25.8)
Registered apprentice or other trade		9 (8.7)		1 (5.0)	10 (8.1)
College, CEGEP, or other nonuniversity		37 (35.6)		4 (20.0)	41 (33.1)
University, without a university degree					
University, with a university degree		6 (5.8)		2 (10.0)	8 (6.5)
Other		25 (24.0) 1 (1.0)		2 (10.0) 1 (5.0)	27 (21.8) 2 (1.6)
Annual household income	99		18		
Less than $10,000		1 (1.0)		2 (11.1)	3 (2.6)
$10, 000–$19,999		6 (6.1)		3 (16.7)	9 (7.7)
$20,000–$39,999		11 (11.1)		3 (16.7)	14 (12.0)
$40,000–$79,999		35 (35.4)		5 (27.8)	40 (34.2)
$80,000 and over		46 (46.5)		5 (27.8)	51 (43.6)
If respondent had ever used CAM	104		22		
Yes (%)		69 (66.3)		14 (63.6)	83 (65.9)

*n*: number with valid responses.

*denotes statistical significance *P* < 0.05; all other *P* values were not significant.

**Table 2:** Commonly used products/practices and their perceived helpfulness.

Product	Ever used *N* (%)	Current use *N* (%)	Perceived helpfulness			
	Total *N* = 74	Total *N* = 46	*n*	Yes *N* (%)	No *N* (%)	Maybe *N* (%)
Vitamins and minerals						
Multivitamin	64 (86.5)	33 (71.7)	55	31 (56.4)	2 (3.6)	22 (40.0)
Folic acid	7 (9.5)	2 (4.3)	5	3 (60.0)	0 (0)	2 (40.0)
Vitamin B	8 (10.8)	1 (2.2)	5	4 (80.0)	0 (0)	1 (20.0)
Vitamin C	32 (43.2)	15 (32.6)	27	19 (70.3)	0 (0)	8 (29.6)
Calcium	17 (23.0)	8 (17.4)	10	10 (100.0)	0 (0)	2 (20.0)
Herbals						
Echinacea	15 (20.3)	5 (10.9)	12	10 (83.3)	0 (0)	2 (16.7)
Garlic	7 (9.5)	2 (4.3)	4	3 (75.0)	0 (0)	1 (25.5)
Peppermint	6 (8.1)	3 (6.5)	5	4 (80.0)	0 (0)	1 (20.0)
Homeopathics						
Cold remedy	21 (28.4)	4 (8.7)	15	14 (93.3)	0 (0)	1 (6.7)
Colic remedy	12 (16.2)	0 (0.0)	8	7 (87.5)	1 (12.5)	0 (0)
Ear drops	13 (17.6)	0 (0.0)	9	8 (88.9)	0 (0)	1 (11.1)
Teething remedy	19 (27.5)	2 (4.3)	14	13 (92.9)	0 (0)	1 (0.71)
Miscellaneous						
Probiotics	9 (12.2)	6 (13.0)	8	6 (75.0)	0 (0)	2 (25.0)
Fish oil/omega 3s	8 (10.8)	2 (4.3)	3	1 (33.3)	0 (0.0)	2 (66.6)
*Practice *	*Total N* = 47	*Total N* = 36				
Aromatherapy	9 (19.1)	5 (13.9)	6	6 (100.0)	0 (0)	0 (0)
Chiropractic	13 (27.7)	5 (13.9)	12	9 (75.0)	2 (16.7)	1 (8.3)
Energy healing	9 (19.1)	3 (8.3)	8	7 (87.5)	0 (0)	1 (12.5)
Faith healing	24 (51.0)	18 (50.0)	24	19 (79.2)	0 (0)	5 (20.8)
Massage	22 (46.8)	14 (38.9)	19	19 (100.0)	0 (0)	0 (0)
Relaxation	12 (25.5)	9 (25.0)	11	11 (100.0)	0 (0)	0 (0)

**Table 3:** Conventional and CAM concurrent use.

Therapeutic agent	No. of users (*n* = 20)	CAM products	Frequency
Immunosuppressants: 6-MP, methotrexate, vincristine, doxirubin, cyclosporine, and prednisone (corticosteroid)	17 (85.0%)	Vitamins/minerals Herbal Homeopathic Probiotics Other	12 1 2 1 1

Antibiotics: Septra, amoxicillin	11 (55.0%)	Vitamins/minerals Herbal Probiotics	8 1 1

Other: dapsone, G-CSF (neutrofil growth factor), gravol (antiemetic), diltiazem (Ca channel blocker), and kayexalate (hyperkalemia)	4 (20.0%)	Vitamins/minerals Other*	3 1

*Other: senecot, BFL chronic fatigue.

## Abbreviations

CAM: Complementary and alternative medicine
CHEO: Children's Hospital of Eastern Ontario
NHP: Natural health product.
